# Arginase as a Critical Prooxidant Mediator in the Binomial Endothelial Dysfunction-Atherosclerosis

**DOI:** 10.1155/2015/924860

**Published:** 2015-05-04

**Authors:** Luiza A. Rabelo, Fernanda O. Ferreira, Valéria Nunes-Souza, Lucas José Sá da Fonseca, Marília O. F. Goulart

**Affiliations:** ^1^Laboratório de Reatividade Cardiovascular, Setor de Fisiologia e Farmacologia, Instituto de Ciências Biológicas e da Saúde (ICBS), Universidade Federal de Alagoas (UFAL), Avenida Lourival Melo Mota s/n, Cidade Universitária, 57072-900 Maceió, AL, Brazil; ^2^Instituto Nacional de Ciência e Tecnologia em NanoBiofarmacêutica (N-BIOFAR), Avenida Antônio Carlos s/n, Pampulha, 31270-901 Belo Horizonte, MG, Brazil; ^3^Max-Delbrück-Center for Molecular Medicine, Robert-Rössle-Str. 10, 13125 Berlin, Germany; ^4^Instituto de Química e Biotecnologia, Universidade Federal de Alagoas (UFAL), Avenida Lourival Melo Mota s/n, Cidade Universitária, 57072-900 Maceió, AL, Brazil

## Abstract

Arginase is a metalloenzyme which hydrolyzes L-arginine to L-ornithine and urea. Since its discovery, in the early 1900s, this enzyme has gained increasing attention, as literature reports have progressively pointed to its critical participation in regulating nitric oxide bioavailability. Indeed, accumulating evidence in the following years would picture arginase as a key player in vascular health. Recent studies have highlighted the arginase regulatory role in the progression of atherosclerosis, the latter an essentially prooxidant state. Apart from the fact that arginase has been proven to impair different metabolic pathways, and also as a consequence of this, the repercussions of the actions of such enzyme go further than first thought. In fact, such metalloenzyme exhibits direct implications in multiple cardiometabolic diseases, among which are hypertension, type 2 diabetes, and hypercholesterolemia. Considering the epidemiological repercussions of these clinical conditions, arginase is currently seen under the spotlights of the search for developing specific inhibitors, in order to mitigate its deleterious effects. That said, the present review focuses on the role of arginase in endothelial function and its participation in the establishment of atherosclerotic lesions, discussing the main regulatory mechanisms of the enzyme, also highlighting the potential development of pharmacological strategies in related cardiovascular diseases.

## 1. Introduction

Cardiovascular and metabolic diseases have achieved global emphasis and represent one of the main public health problems [1–3]. The augmented burden of such conditions is due to the increase of their risk factors in alarming epidemic proportions, becoming an important cause of morbidity and mortality in occidental countries. In this scenario, atherosclerosis is recognized as a hallmark in the development of key cardiovascular disorders, including myocardial infarction and stroke [3–5].

Thereby, many studies have aimed to elucidate the pathophysiological mechanisms involved in the onset and development of these diseases, highlighting the role of endothelial dysfunction in vascular disorders, which is mainly caused by the reduced bioavailability of nitric oxide (^•^NO; ^•^N=O nitrogen monoxide) [6]. When it comes to the ^•^NO bioavailability, it is of note to emphasize the role of the semiessential amino acid L-arginine, a common substrate for both nitric oxide synthase (NOS) and arginase enzymes [7].

In a pioneer work, Buga and coworkers showed that the NG-hydroxy-L-arginine, an intermediate compound in the process of  ^•^NO synthesis from L-arginine, is an endogenous inhibitor of arginase activity [8]. In this context, it becomes evident the important role of these metabolic enzymes, with the necessity to keep the balance for the axis NOS/L-arginine/arginase for maintaining the ^•^NO homeostatic levels. The two fundamental mechanisms for reduced levels of bioactive ^•^NO are its reduced synthesis by NOS and its increased oxidative inactivation by reactive oxygen species (ROS) intermediates, ultimately leading to a potential impairment in cardiovascular homeostasis [9].

Mammalian arginase (EC 3.5.3.1), a manganese-metalloenzyme [10], hydrolyzes L-arginine to L-ornithine and urea [11].

By presenting this action on L-arginine, arginase participates in the regulation of the ^•^NO synthesis by using the same enzyme substrate for the three known NOS isoforms: neuronal NOS (nNOS or NOS-1), inducible NOS (iNOS or NOS-2), and endothelial NOS (eNOS or NOS-3) [12]. Thus, increased expression of arginase may imply extensive consumption of L-arginine to be converted into urea and L-ornithine, this way reducing the availability of L-arginine to ^•^NO synthesis by NOS.

**Figure 1:**
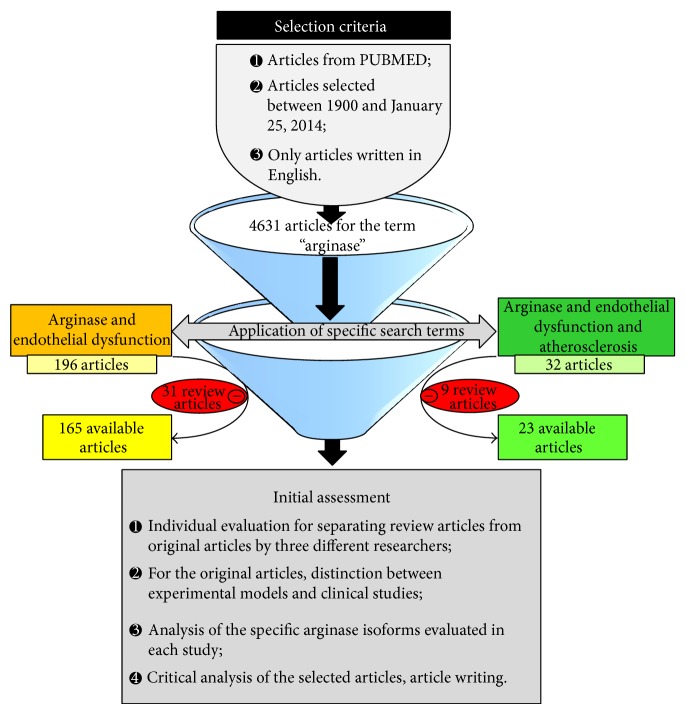
Workflow: steps for articles selection and for preparing the paper.

Various studies have demonstrated increase in arginase activity in different clinical conditions, such as hypertension [13, 14], type 2 diabetes mellitus [15, 16], hypercholesterolemia [17], aging [18–20], and atherosclerosis [21], proposing a critical contribution of this enzyme in the pathogenesis of cardiovascular diseases. In this direction, the present review focuses on the role of arginase in atherosclerosis and its implications in endothelial function, aiming to contribute to the pathophysiological discussion of the main regulatory mechanisms of the enzyme. Also, the paper highlights the potential development of pharmacological actions in related cardiovascular diseases concerning the arginase activity. For carrying out this study, a research in electronic PubMed database was performed taking as reference the period between the year 1900 and January 25, 2014, using the terms “arginase AND endothelial dysfunction,” and “arginase AND endothelial dysfunction AND atherosclerosis.” Only articles available in English were considered (Figure 1). In the following lines, the term “arginase” will be used to refer to both isoforms of the enzyme, so that arginase I will be specifically presented by the term “Arg I,” and the second one, by “Arg II.”

## 2. From the Discovery of Arginase to Its Role in Endothelial Dysfunction: More Than a Century of History

The seminal events of the history of arginase took place more than a hundred years ago. In 1904, Kossel and Dakin [22] described the discovery of this enzyme in mammalian liver. They observed a decrease of arginine after acid hydrolysis in the liver caused by administration of the ferment named arginase, generating urea and ornithine [11, 22]. Thenceforth, other researches have reported the presence of arginase in various organs. In the first decades of 1900, arginase was found in the liver of several animal classes such as amphibians, fishes, and turtles by Clementi, who also identified the presence of the enzyme in the kidney of birds.

A few years after Clementi's observations [23–25], the records concerning the spectrum of arginase distribution in different organs of certain mammals and domestic fowls were broadened by Edlbacher and Rothler [26], as both authors conducted a large study which brought to light the identification, in mammals and birds, of arginase in the liver, kidneys and testes. Besides showing that the number of units of arginase normalized by body weight differed between sexes, being elevated in males when compared to females, the authors also reported the presence of arginase in the placenta and thymus of some mammals. Later on, in 1927, Chaudhuri performed a study in 32 birds showing a quantitative estimation of arginase mainly in kidneys and then in testes. He also defended a gender distinction in the arginase distribution due to the presence of this enzyme in male sexual organs and the absence in female ones [27]. In the following years, the differential role of arginase between sexes and the participation of sex steroid hormones in its modulation would also be discussed, pointing to the possible effects of arginase in multiple pathophysiological pathways [28].

In 1930, it was reported the activation of arginase by metal complexes of thiols, such as reduced glutathione and Fe^+2^ and Cu^+2^ ions. However, Purr and Weil [29] suggested that a specific oxidation-reduction potential, and not only the SH group, was related to arginase activation [29]. In 1986, Dizikes and coworkers published the screening of human liver arginase cDNA, which was not completely homologous to the genes found in the human kidney [30]. Such apparent discrepancy was posteriorly ruled out when the sequence found in the kidney was described as the gene of Arg II, cloned in 1996 [31]. Throughout the years, several studies have demonstrated the presence of arginase not only in various organs and species but also in animal models related to vascular disorders [20, 21, 32–38]. Buga and coworkers, for example, demonstrated for the first time the constitutive expression of arginase in rat aortic endothelial cells, also observing that its endogenous inhibition may represent a means for enabling the availability of proper amounts of arginine for ^•^NO production [8]. Furthermore, by comparing young and old Wistar rats, Berkowitz and coworkers first showed the association between arginase activity and the endothelial dysfunction of aging [20]. Following these breakthroughs in the comprehension of arginase contribution to vascular health, Ryoo and coworkers [21] discussed the connections among atherosclerosis, arginase activity, and endothelial dysfunction in atherosclerosis-prone mice, highlighting the therapeutic potential of arginase in atherosclerotic vascular disease [21]. Nevertheless, the pathophysiological mechanisms concerning the arginase participation in different metabolic diseases are not completely understood and the researches in humans still require further advances in order to better define the molecular pathways through which arginase interferes in health and disease.

## 3. In the Backstage of the Enzyme Function: Expression, Regulation of Arginase, and the Crosstalk in the Signaling Pathways

Arginase is an enzyme that participates in the urea cycle, being described in two isoforms: Arg I and Arg II, both of them catalyzing the same biochemical reaction. Human Arg I is a 322-amino acid protein, sharing 58% sequence with her sister, Arg II [39]. Arg I is known as the hepatic isoform because it is primarily found in the liver [40, 41]. However, it has been described in other tissues such as endothelial cells and vascular smooth muscle cells (VSMC), being also a cytosolic enzyme [42]. On the other hand, in mammalians, Arg II is a mitochondrial enzyme and is distributed in several organs and tissues, including prostate, kidney [39], and blood vessels. Their isoforms are expressed in the vasculature, as well as have many actions that interfere with vascular dynamics and contribute to endothelial dysfunction found in various cardiovascular pathologies [21].

In the course of the enzyme activity, the hydrogen bonding established between the guanidinium group and Glu227 keeps L-arginine in its proper position/location in the active site of the enzyme. Such molecular interaction is critical for enabling L-arginine to be attacked by the metal-associated hydroxide ion at the guanidinium group, leading to the formation of a tetrahedral intermediate. Thus, the hydroxyl group in that intermediate and the developing sp^3^ lone electron pair on the NH_2_ group are stabilized by the manganese ions [10].

According to the aforementioned statements, in the endothelial layer, the competition between Arg II and eNOS leads to a decrease in the ^•^NO bioavailability, resulting in impaired vasodilation and, consequently, endothelial dysfunction [20, 21]. The consumption of L-arginine also stimulates the production of reactive oxygen species (ROS), greatly contributing to oxidative stress. In addition, arginase induces the synthesis of polyamines and proline, promoting VSMC proliferation and remodeling [43, 44]. It has also been reported that the overexpression of Arg I reduced inflammatory activity in rabbits, via interaction with endothelial nitric oxide synthase (eNOS) [45]. Furthermore, it has been reported that conditions such as hypoxia [46], as well as proinflammatory mediators [47], reactive oxygen and nitrogen species (RONS), glucose, and oxidized low-density lipoprotein (ox-LDL) [40] stimulate arginase expression. Together, factors including stimulation and expression of this enzyme provide a framework for the analysis of the arginase isoforms into the atherogenesis. In endothelial cells, the abovementioned inducing phenomena are particularly of note, once the reduction in L-arginine levels is presented as one of the main mechanisms which may lead to the establishment of endothelial dysfunction [45]. Accordingly, several signaling pathways involve the activation of arginase, such as receptors for ox-LDL [21, 48], inflammatory mediators, and RhoA/ROCK kinases [45]. At first, after the oxidation of LDL, the resulting lipoprotein binds to the lectin-like LDL receptor-1, LOX-1, stimulating the activation of arginase [21]. Furthermore, ox-LDL increases the expression of caveolin I, a molecule that interacts with eNOS, hindering the formation of ^•^NO [48, 49].

With regard to inflammatory mechanisms, macrophages, cells involved in the generation of the atheromatous plaque, induce the expression of arginase via lipopolysaccharide (LPS), interleukin (IL)-4, IL-6, and interferon-gamma (INF-*γ*) [45]. In addition, in inflammatory conditions, cationic amino acid transporters (CAT), which act as L-arginine transporters, have their function impaired, then reducing the production of  ^•^NO and consequently promoting the progression of atherosclerosis [50]. Moreover, the route RoA/ROCK corresponds to a cascade of intracellular signaling through which protein kinases, stimulated by various factors, such as proinflammatory cells, ROS, and ox-LDL, induce the activation of arginase [45, 51].

## 4. Vascular Dysfunction in Atherosclerosis: A Brief Update

The endothelium is a central component for the maintenance of cardiovascular homeostasis. For a long time, it was thought that the vascular endothelium predominantly would act as a surface for blood flow. In a landmark study, Furchgott and Zawadzki [52] changed this concept. These authors first demonstrated the existence of an endothelium-derived relaxing factor which was subsequently identified as ^•^NO [53–55]. Further work showed that the endothelium is an active participant in the regulation of cardiovascular homeostasis [56]. The network involved in the endothelial regulation is complex. Under physiological conditions, this cell layer is a sensor of hemodynamic changes and releases both relaxing and contracting factors. The disturbance in this sensitive balance leads to endothelial dysfunction, a common feature in cardiovascular, renal, and metabolic diseases [57], as well as in the atherosclerotic plaque formation [4, 58].

Atherosclerosis represents a multifactorial process and one of the well discussed marks of this structural and functional phenomenon is the role of cholesterol and inflammatory mediators in atheroma formation [3, 5]. Currently, it is defined as a chronic and progressive disease in which the deposition of atherosclerotic plaques occurs on the inner face of great arteries, causing reduction of the vascular lumen and damage to the underlying layers [3, 4].

The initial step in the formation of an atherosclerotic plaque is characterized by damage, either structural or functional, in the inner surface of the arteries, which promotes the increased expression of adhesion molecules in the surface of endothelial cells, thereby stimulating leukocyte adhesion to the intima layer. In the establishment of this process, proinflammatory mediators and shear stress play an important role, favoring this adherence. After physically contacting the endothelial cells, the adhered monocytes may infiltrate the subendothelial space via migration. Concomitantly, an accumulation of LDL particles in that space may also occur. In the presence of oxidative stress, the increased amounts of ROS, among which superoxide anion (^•^O_2_^−^), lead to the oxidation of such lipoproteins (ox-LDL). The circulating monocytes, now recruited and turned into macrophages in the subendothelial space, progressively phagocyte ox-LDL particles, ultimately being converted in foam cells. In this process, VSMC are stimulated to migrate from the tunica media to the intima, producing a cape of collagen and elastin that covers the plaque. Additionally, it is also observed the formation of a necrotic core due to debris and lipids released from cells that suffered apoptosis [5, 59].

Experimental and clinical evidence show that the major risk factors implicated in the impairment of cardiovascular functions are directly associated with endothelial dysfunction [56, 60, 61]. The seminal experiments of Ludmer and coworkers [58], using angiography, characterized the paradoxical effect of acetylcholine (ACh) in the coronary circulation, as this study showed that, in patients with atherosclerosis, this muscarinic agonist significantly decreased vascular diameter. The authors postulated that the impairment in endothelial function is responsible for vasoconstriction observed after intracoronary administration of ACh [58].

The link between endothelial cells and the elementary changes in the vascular wall which precede the establishment of the atherosclerotic process has passed from drafted speculations to concrete evidences, once some studies have clearly shown the connection between endothelial dysfunction and the early development of atherosclerosis [5, 45]. In order to elucidate the relationship between ^•^NO and endothelial dysfunction in atherosclerosis, for example, Dhawan and colleagues [62] demonstrated a decrease in atherosclerotic plaque formation in an experimental model with monkeys fed a high-cholesterol diet after administration of L-arginine, with improved endothelial function by elevation of  ^•^NO levels and thereby reduced atherogenesis. In this context, several studies have been conducted to characterize the relationship between arginase and the cardiovascular endpoint of progressive endothelial dysfunction, marked by atherosclerosis.

Taking into account that the regulation in ^•^NO metabolism represents a mediator for vascular health [63] and that arginase activity in particular conditions may play “the bad guy” by compromising the ^•^NO bioavailability [7, 9], it becomes easy to understand the reason why the ^•^NO/arginase axis is now recognized as a pivotal regulatory pathway of the vascular system. Indeed, for arginase activity there is only one substrate, L-arginine, but the repercussions of this single action may reach massive proportions, implying pleiotropic harmful outcomes on the endothelial function, once the beneficial effects of ^•^NO, among which the prevention of abnormal vasoconstriction, inhibition of platelets aggregation, and reduced expression of adhesion molecules in the surface of endothelial cells [64], could be nullified.

## 5. Role of Arginase in the Development of Endothelial Dysfunction and Atherosclerosis

Studies have demonstrated an increase in the regulation of arginase in various cardiometabolic diseases such as hypertension [65–67], atherosclerosis [15, 21], ischemia-reperfusion injury [36, 68], diabetes mellitus [15, 57], and ageing [20, 69]. These findings have prompted researchers to discover whether arginase inhibition would then result in improved endothelial function in these conditions.

**Figure 2:**
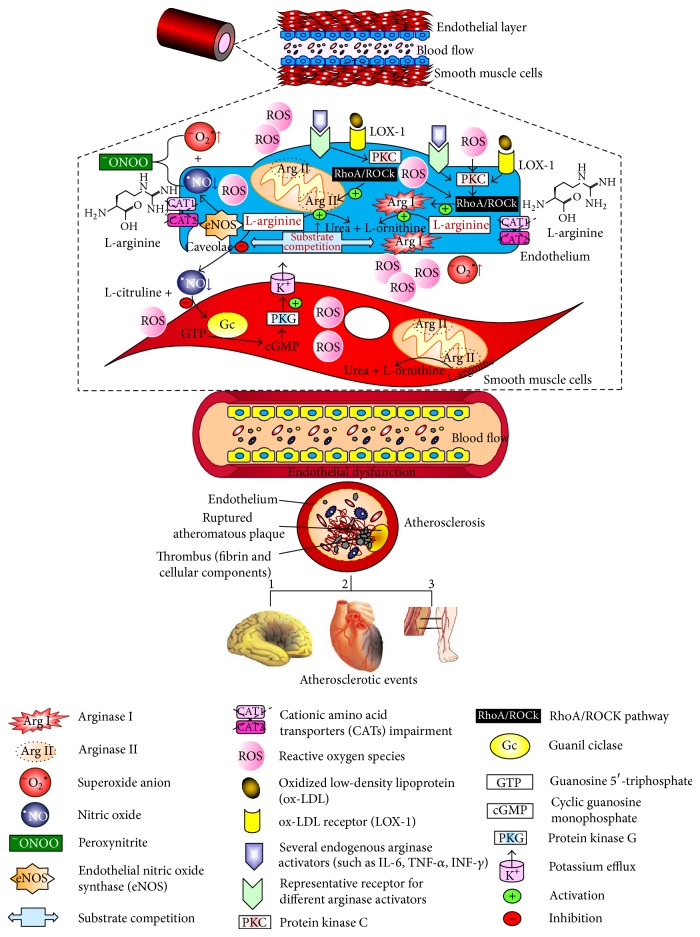
Role of arginase isoforms in nitric oxide metabolism in the vasculature. The complex web of interactions among circulating factors, membrane receptors, and intracellular signaling pathways directly interferes with vascular homeostasis. The balance between the activities of the endothelial nitric oxide synthase and the arginase isoforms is critical for maintaining the adequate nitric oxide bioavailability. Once the imbalance is established, either for increased reactive oxygen species production, decreased nitric oxide availability, or both, the phenomenon of endothelial dysfunction (in this figure represented by endothelial cells in yellow) may occur, being the initial event in the establishment and progression of atherosclerosis. As a consequence of such vascular damage, the arterial impairment progresses, increasing the risk of developing different atherosclerotic events, among which are stroke (1), myocardial infarction (2), and peripheral artery disease (3).

As previously discussed, a decrease of the ^•^NO bioavailability plays an essential role in the pathogenesis of various cardiovascular events [70]. Interestingly, endothelial dysfunction derived from the ^•^NO reduction causes vascular stiffness even in the absence of atherosclerosis, with arginase presenting itself as a key element in the progression of vascular disorders [71] (Figure 2).

Arginase acts in atherogenesis mostly via reduction of ^•^NO, a free radical responsible for the inhibition of platelet aggregation and leukocyte adhesion to the blood vessel wall, being an important factor in the formation of atherosclerotic plaque [5]. Furthermore, ^•^NO helps in the preservation of endothelial function due its vasodilator effect, so that its deficiency corresponds to another mark that leads to the progression of atherosclerosis [72] (Figure 2). Ryoo and coworkers [21], while studying the role of Arg II in atherosclerosis, found that ox-LDL, frequently observed in this disease, stimulates the release of Arg II, reducing the production of ^•^NO. In such work, the researchers used mice with genetic deletion for Arg II (Arg II^−/−^) fed a high-cholesterol diet, observing that the reduction in arginase activity improved the endothelial function compared to ApoE^−/−^ mice fed the same diet. These findings suggest that the genetic deletion of arginase provides endothelial protection. Another crucial atherosclerotic pathophysiological mechanism consists in the oxidation of LDL particles [40, 48], that contributes to the formation of foam cells, after being captured by phagocytes. This also participates in the formation of ROS via NADPH oxidase [73] and uncoupling of eNOS [40], hindering the ^•^NO production. In addition, it was shown that hypercholesterolemia increases the generation of asymmetrical dimethyl-L-arginine (ADMA), an endogenous inhibitor of eNOS [9, 64], in experimental models with monkey, inhibiting competitively the binding of L-arginine to eNOS [74].

ox-LDL causes a separation of arginase from the microtubule cytoskeleton, then increasing the expression of this enzyme in human aortic endothelial cells (HAECs) [40]. Ryoo and colleagues [48] showed that the isoform in question corresponds to Arg II via LOX-1 receptor. Furthermore, as a consequence of arginase activity in the formation of the atherosclerotic plaque, it is observed a reduction in ^•^NO levels, thereby inducing the oxidation of LDL (which generates a stimulatory form of atherogenesis) and other effects such as increased endothelial permeability, cell proliferation, and leukocyte adhesion on the vasculature, resulting in atherosclerosis [75].

Several studies have reported endothelial dysfunction as a consequence of increased activity and/or expression of arginase in experimental models of hypertension [70], diabetes mellitus [15], aging [20], erectile dysfunction, sickle cell disease, and atherosclerosis [70], the latter corresponding to the focus of this paper. In Arg II null mice, for example, this enzyme activity was substantially reduced in the vascular endothelium, suggesting that Arg II is the main isoform present in blood vessels. Also, the inhibition of Arg II restored endothelial function, led to increased vascular ^•^NO levels and decreased vascular stiffness in ApoE^−/−^ mice [21]. Moreover, even in ApoE^−/−^ mice, the high-cholesterol diet increased arginase activity compared with control mice fed a standard diet. In addition, inhibition of Arg II prevented the ^•^NO decrease induced by high-cholesterol diet [21].

The rise of RONS due to the increased expression of arginase has emerged as one of the factors that induce endothelial dysfunction in animal models of atherosclerosis [41], a fact that has also been observed in patients with coronary artery disease and vascular impairment in type 2 diabetes mellitus [76]. However, it is not completely understood how arginase acts on endothelial dysfunction* in vivo* [15].

An established concept, however, defines that the production of RONS is also stimulated by inflammatory conditions, leading to endothelial injury and consequent development of atherosclerosis [50].

The formation of ROS is reported as a hallmark in the physiopathology of atherosclerosis. In this regard, the oxidative modification hypothesis as a critical step in the development of atherosclerosis deserves to be mentioned. According to this hypothesis, the simple presence of circulating LDL particles is not the only initial influencing factor for the onset of atheromatous plaques. Instead, such particles are required to undergo structural changes so that they can be properly recognized by specific macrophage receptors, being then engulfed and accumulated in phagocytes [77, 78]. In line with these observations, several studies have demonstrated the role of ox-LDL and proinflammatory mediators in the generation of atheromatous plaque [5]. One of the mechanisms arising from these stimuli corresponds to apoptosis of endothelial cells, which contributes to the induction of atherogenesis. Given this context, Suschek and coworkers [50] demonstrated that the expression of IL-1, tumor necrosis factor alfa (TNF-*α*), and INF-*γ* in blocked iNOS rat models resulted in cell apoptosis induced by hydrogen peroxide (H_2_O_2_), whereas under conditions of high levels of ^•^NO, no protection against cell death was observed, concluding that a greater supply of L-arginine helps to reduce the development of atherosclerosis [50].

As previously outlined, endothelial dysfunction and atherosclerosis are directly linked to proinflammatory states in blood vessels. Several cytokines, such as IL-4, IL-6, and TNF stimulate the activity of arginase and impair the expression of eNOS [79], besides increasing the production of ROS (Figure 2). Concerning this issue, Spillman and colleagues [80] investigated the relationship between liver X receptors (LXR), a hormone receptor that participates in the reverse transport of cholesterol and TNF upregulation, demonstrating a decrease in Arg II activity and mRNA expression by LXR agonist, with restoration of  ^•^NO bioavailability.

In another study focused on inflammatory factors involved in endothelial dysfunction, Witting and coworkers [81] showed an increase in arginase protein expression and activity in rat aorta exposed to protein serum amyloid A (SAA), an apolipoprotein produced by the liver that is deposited in atherosclerotic plaques and is costimulated by inflammatory mediators such as TNF-*α*, IL-1, and IL-6. Reinforcing the critical role of arginase in the metabolism of ^•^NO, experiments conducted by Sikka and coworkers [12] showed that, in C57Bl/6 mice exposed to cigarette smoke for 2 weeks, the knockout mice for Arg II showed better endothelial function compared to the controls. This observation was attributed to the gene deletion of Arg II and the consequent increase in ^•^NO bioavailability [12] (Figure 2).

One of the mechanisms that lead to endothelial dysfunction is characterized by shear stress, reported as a predisposing factor for plaque formation. In a novel study with porcine endothelial cells and carotid artery, Thacher and colleagues [82] demonstrated an increase of Arg II expression after the induction of oscillatory shear stress compared to unidirectional high shear stress, for three days. To corroborate the role of this enzyme with the related alteration in the blood flow, the arginase inhibitor N^w^-hydroxy-nor-L-arginine (Nor-NOHA) was administered, resulting in a decrease in ROS production [82]. Furthermore, an elevated proliferation of VSMC was also observed. With regard to this effect, Xiong and coworkers [83] studied the role of Arg II in VSMC from human umbilical veins, also demonstrating a proliferative action in these cells when the enzyme was activated. However, the authors observed an induction to senescence and apoptosis in the lack of Arg II function, contributing to the rupture of the plaque burden, due to the resulting weakness of the vascular layers [83] (Figure 2).

With regard to arginase stimulation, the administration of thrombin in human umbilical vein cells was able to elevate this enzyme expression after 18 hours of exposure, with peak in 24 hours [84]. In this study, it was also administered the HMG-CoA inhibitor fluvastatin, which impairs the RhoA/ROCK pathway, leading to reduction of arginase expression by thrombin. In addition, similar effects were observed with other ROCK inhibitors used in the study [84]. Also, while studying the role of Arg II in the macrophage inflammatory responses, Ming and coworkers found a protective profile against insulin resistance, type 2 diabetes, and atherosclerosis in Arg II-deficient mice [38]. In addition, by comparing the vascular function between transgenic C57Bl/6 mice overexpressing human Arg II and nontransgenic controls, Vaisman and colleagues [70] found impaired endothelium-dependent vasodilation induced by ACh in the transgenic group. The authors also observed that the increased expression of Arg II itself, irrespective of changes in lipid concentrations in plasma, was sufficient to feed the development of atherosclerotic lesions, ultimately highlighting the critical role of Arg II in such inflammatory process.

Aside from being described as a potentially deleterious enzyme in the regulation of endothelial function, of great importance are also the observations that arginase may exert beneficial effects in the vasculature. Such capacity of, in specific circumstances, embodying both a protective and harmful role, inserts arginase in an even more complex and intriguing context. In rabbits, for example, while studying genes of atherosclerosis susceptibility, Teupser and coworkers [85] showed that the high expression of Arg I in macrophages contributes to atherosclerosis resistance, possibly by exerting an anti-inflammatory mechanism in the vascular environment [85]. Furthermore, the atheroprotective role of Arg I may be expressed through multiple pathways, such as differential activation of macrophages, modulation of inflammatory response in VSMC, and changes in plaque stability [41].

All original studies assessed in the present paper, concerning the role of arginase isoforms in the binomial endothelial dysfunction-atherosclerosis, are presented in Table 1.

## 6. The Therapeutic Potential of Arginase: A Short Update

Arginase inhibitors have been developed since the 1990s, to evaluate more accurately the effects of the enzyme activity reduction [86], with the observation of promising results concerning the improvement in endothelial function and associated disorders in animal models of diabetes [34], hypercholesterolemia [87], hypertension [32, 37, 88], and metabolic syndrome [88].

Therefore, the elucidation of mechanisms involved in the transcription and transduction, as well as in activity of arginase represents a promising area for the development of pharmacological approaches in cardiovascular and metabolic diseases [45, 86]. Despite the growing number of studies and the evident role of arginase in the regulation of ^•^NO bioavailability [8, 68, 89, 90] and endothelial dysfunction [20, 33, 91], development of atherosclerosis [21, 40], and other cardiometabolic diseases [34, 37, 57, 92], several questions still need to be better understood.

In fact, a major obstacle for a more accurate understanding of the effects of the two different human arginases still rests on the difficulty in creating a specific inhibitor for each isoform, due to the close similarity of chemical structure between them, which contains almost identical metal clusters and active site configuration [86]. In addition, the nonspecificity of the inhibitors becomes a challenge in studies with animals, as well as for future clinical trials, because the unspecific inhibition would not enable to define for sure to which isoform the observed effects could be attributed.

In this scenario, despite the encouraging findings concerning enzyme inhibition, the use of arginase inhibitors still claims for advances, since the pharmacological agents currently available are not isoform-specific. This fact is of particular importance, considering the differential organic distribution, concentrations, and specific actions of arginase isoforms, as stated above.

So far, different studies have already pointed to the potential of arginase inhibition, either directly or indirectly. In rats presenting with metabolic syndrome treated with arginase inhibitors (citrulline, norvaline, and ornithine), for example, it was observed amelioration in blood pressure levels directly by increase in ^•^NO bioavailability and indirectly by inhibition of insulin resistance and hypertriglyceridaemia [88]. Also, Holowatz and Kenney demonstrated that essential hypertension in humans was associated with attenuated reflex cutaneous vasodilation and that acute inhibition of arginase improved such reflex [93]. Similarly, oral administration of atorvastatin for three months was capable of restoring cutaneous microvascular function in hypercholesterolaemic patients, with the improvement observed mediated by decreased arginase activity [94]. Taken together, these observations highlight the multiple ways through which arginase may be targeted in order to improve cardiometabolic profiles.

## 7. Conclusions and Perspectives

In summary, increases in arginase expression and/or activity undoubtedly repercute in the atherosclerosis physiopathology, mainly by regulating the ^•^NO production via eNOS, so that the upregulation of arginase favors the formation of the atherosclerotic plaque [95].

Thus, further studies are required to explain the degree with which arginase contributes in the intrinsic mechanisms of endothelial dysfunction, principally in the clinical setting. In this regard, clinical research is still limited, with few human studies that assessed the role of arginase in atherosclerosis. Indeed, by allowing the dissection of the molecular and metabolic pathways trodden by the actions of such enzyme, this investigation represents a promising field for developing new targeted therapies for different clinical conditions.

Finally, considering the amount of knowledge that has been accumulated in the last decades, pointing to the well-recognized participation of arginase in the onset and development of endothelial dysfunction and cardiovascular diseases, a myriad of fresh perspectives under the molecular point of view was opened widely right before our eyes. If in 1927, Chaudhuri would say that “*As regards the function of the enzyme, very little is as yet known except that it hydrolyses arginine into ornithine and urea*” [27], the increasing attention given to that newborn and mysterious enzyme in the following years would change that statement dramatically. The pieces of this puzzle were randomly spread on Kossel and Dakin's table, inciting the curiosity of other pioneer researchers in the study of arginase from the first half of the twentieth century. It is our work, from now on, to keep on putting them back together, once recent advances in the study of the triad arginase/endothelial dysfunction/atherosclerosis have shown that these pieces, little by little, have finally began to fit properly with each other. It is not possible to foresee what the future might hold, but it seems to be just a matter of time for us to evidence so many other hidden connections linking the paths of this journey and learn as much as possible from what they can tell us.

## Figures and Tables

**Table 1:** Original articles approaching the arginase isoforms in different clinical and experimental studies.

Arginase actions and their responses in the vasculature					
Reference	Type of study	Study design	Type of arginase involved	Actions/results	Vascular damage
[12]	Preclinical	*In vivo* study with *postmortem* analysis in wild type C57Bl/6 and arginase II knockout male mouse (assessment of cigarette smoke effects)	Arg II		
[15]	Clinical	*In vivo *study performed in patients with coronary artery disease and type 2 diabetes mellitus	Arg I and Arg II		
[21]	Preclinical	*In vivo *study with male ApoE^−^/^−^ and C57Bl/6 mouse and *postmortem* analysis	Arg II		
[70]	Preclinical	*In vivo* model of transgenic C57Bl/6 mouse overexpressing human arginase II, with *postmortem* analysis	Arg II		
[82]	Preclinical	*In vitro* study in cell culture of isolated porcine carotid endothelium	Arg II		
[96]	Clinical	Clinical study with blood samples of patients with type 2 diabetes mellitus	Arg I and Arg II		
[97]	Preclinical	*In vitro* study using isolated mouse aortic endothelium and HUVECs cultures	Arg II		
[6]	Clinical	Human popliteal and tibial vessels from amputation specimens (*ex vivo* model)	Arg II	Reduction of ^•^NO bioavailability	Endothelial dysfunction
[48]	Preclinical	*In vivo* and *in vitro* study using culture of human aortic endothelial cells (HAECs) and C57Bl/6 mouse aortic rings	Arg II		
[50]	Preclinical	*In vitro* study performed on rat aorta endothelial cells (AECs)	Arg I and Arg II		
[80]	Preclinical	*In vitro* study performed on HUVEC and Wistar rat aortic rings	Arg II		
[81]	Preclinical	*In vitro* study performed on culture of human aortic endothelial cells and Wistar rat aortic rings	Arg I and Arg II		
[95]	Preclinical	*In vitro* study with treatment in HUVECs and mouse aortic rings	Arg I and Arg II		
[98]	Preclinical	Study performed on HUVECs culture *in vitro* and isolated C57Bl/6 mouse aorta	Arg I and Arg II		
[69]	Preclinical	*In vitro *study using HUVECs culture and *in vivo* treatment in C57Bl/6 mice	Arg I and Arg II		
[12]	Preclinical	*In vivo* study with *postmortem* analysis in C57Bl/6 and arginase II knockout male mice (assessment of cigarette smoke effects)	Arg II	Uncoupling of eNOS	Increased ROS production
[21]	Preclinical	*In vivo *study with male ApoE^−^/^−^ and C57Bl6 mice and *postmortem* analysis	Arg II		
[48]	Preclinical	*In vivo* and *in vitro* study using culture of human aortic endothelial cells (HAECs) and C57Bl/6 mouse aortic rings	Arg II		
[69]	Preclinical	*In vitro *study using HUVECs culture and *in vivo* treatment in C57BL/6 mice	Arg I and Arg II		
[80]	Preclinical	*In vitro* study performed on HUVEC and Wistar rat aortic rings	Arg II		
[97]	Preclinical	*In vitro* study using isolated mouse aortic endothelium and HUVECs cultures	Arg II		
[95]	Preclinical	Study performed on HUVECs culture *in vitro* and isolated C57BL/6 mouse aorta	Arg I and Arg II		
[98]	Preclinical	Study performed on HUVECs culture *in vitro* and isolated C57BL/6 mouse aorta	Arg I and Arg II		
[69]	Preclinical	*In vitro *study using HUVECs culture and *in vivo* treatment in C57Bl/6 mouse	Arg I and Arg II		
[82]	Preclinical	*In vitro* study in cell culture of isolated porcine carotid endothelium	Arg II		
[83]	Preclinical	*In vitro* (VSMCs from human umbilical vein) and *in vivo* (ApoE^−^/^−^ and C57Bl/6 mouse) study	Arg II		

Arg I: arginase I; Arg II: arginase II; eNOS: endothelial nitric oxide synthase; HAECs: human aortic endothelial cells; HUVECs: human umbilical vein endothelial cells; ^•^NO: nitric oxide; ROS: reactive oxygen species; VSMC: vascular smooth muscle cells.

## References

[1] Alberti K. G. M. M., Eckel R. H., Grundy S. M. (2009). Harmonizing the metabolic syndrome: a joint interim statement of the International Diabetes Federation Task Force on epidemiology and prevention; National Heart, Lung, and Blood Institute; American Heart Association; World Heart Federation; International Atherosclerosis Society; and International Association for the Study of Obesity. *Circulation*.

[2] Cameron A. J., Zimmet P. Z., Shaw J. E., Alberti K. G. M. M. (2009). The metabolic syndrome: in need of a global mission statement. *Diabetic Medicine*.

[3] Libby P., Ridker P. M., Hansson G. K. (2011). Progress and challenges in translating the biology of atherosclerosis. *Nature*.

[4] Libby P. (2002). Inflammation in atherosclerosis. *Nature*.

[5] Hopkins P. N. (2013). Molecular biology of atherosclerosis. *Physiological Reviews*.

[6] Lakin R. O., Zhu W., Feiten L., Kashyap V. S. (2013). Techniques to harvest diseased human peripheral arteries and measure endothelial function in an ex vivo model. *Journal of Vascular Surgery*.

[7] Durante W., Johnson F. K., Johnson R. A. (2007). Arginase: a critical regulator of nitric oxide synthesis and vascular function. *Clinical and Experimental Pharmacology & Physiology*.

[8] Buga G. M., Singh R., Pervin S. (1996). Arginase activity in endothelial cells: Inhibition by N(G)-hydroxy-L- arginine during high-output NO production. *American Journal of Physiology—Heart and Circulatory Physiology*.

[9] Harrison D. G. (1997). Cellular and molecular mechanisms of endothelial cell dysfunction. *Journal of Clinical Investigation*.

[10] Ash D. E., Cox J. D., Christianson D. W. (2000). Arginase: a binuclear manganese metalloenzyme. *Metal Ions in Biological Systems*.

[11] Wakeman A. J. (1905). On the hexon bases of liver tissue under normal and certain pathological conditions. *The Journal of Experimental Medicine*.

[12] Sikka G., Pandey D., Bhuniya A. K. (2013). Contribution of arginase activation to vascular dysfunction in cigarette smoking. *Atherosclerosis*.

[13] Xu W., Kaneko F. T., Zheng S. (2004). Increased arginase II and decreased NO synthesis in endothelial cells of patients with pulmonary arterial hypertension. *The FASEB Journal*.

[14] Kaminskiǐ I. G., Suslikov A. V., Tikhonova L. A. (2011). Arginase, nitrates, and nitrites in the blood plasma and erythrocytes in hypertension and after therapy with lisinopril and simvastatin. *Izvestiia Akademii nauk. Seriia biologicheskaia/Rossiǐskaia akademiia nauk*.

[15] Shemyakin A., Kövamees O., Rafnsson A. (2012). Arginase inhibition improves endothelial function in patients with coronary artery disease and type 2 diabetes mellitus. *Circulation*.

[16] Kashyap S. R., Lara A., Zhang R., Young M. P., DeFronzo R. A. (2008). Insulin reduces plasma arginase activity in type 2 diabetic patients. *Diabetes Care*.

[17] Leiva A., de Medina C. D., Salsoso R. (2013). Maternal hypercholesterolemia in pregnancy associates with umbilical vein endothelial dysfunction: role of endothelial nitric oxide synthase and arginase II. *Arteriosclerosis, Thrombosis, and Vascular Biology*.

[18] Biczó G., Hegyi P., Berczi S. (2010). Inhibition of arginase activity ameliorates l-arginine-induced acute pancreatitis in rats. *Pancreas*.

[19] Aldemir D., Tufan H., Tecder-Ünal M. (2003). Age-related alterations of oxidative stress and arginase activity as a response to intestinal ischemia-reperfusion in rat kidney and liver. *Transplantation Proceedings*.

[20] Berkowitz D. E., White R., Li D. (2003). Arginase reciprocally regulates nitric oxide synthase activity and contributes to endothelial dysfunction in aging blood vessels. *Circulation*.

[21] Ryoo S., Gupta G., Benjo A. (2008). Endothelial arginase II: a novel target for the treatment of atherosclerosis. *Circulation Research*.

[22] Kossel A., Dakin H. D. (1904). Ueber die Arginase. *Zeitschrift für Physiologische Chemie*.

[23] Clementi A. (1916). Presenza del fermento ureogenetico nel fegato di embrione umano e suo significato fisiologico. *Atti della Reale Accademia dei Lincei, Rendiconti*.

[24] Clementi A. (1918). Sulla presenza dell' arginasi nell' organism di qualche invertebrati. *Atti della Reale Accademia dei Lincei, Rendiconti*.

[25] Clementi A. (1922). L'arginasi nella mucosa enterica e nel secreto enterico. *Atti della Reale Accademia dei Lincei, Rendiconti*.

[26] Edlbacher S., Rothler H. (1925). Die quantitative Bestimmung der Arginase in tierischen Organen. *Zeitschrift für Physikalische Chemie*.

[27] Chaudhuri A. C. (1927). A study of Arginase content in the fowl with special reference to sex. *The Journal of Experimental Biology*.

[28] Kim N. N., Christianson D. W., Traish A. M. (2004). Role of arginase in the male and female sexual arousal response. *Journal of Nutrition*.

[29] Purr A., Weil L. (1934). The relation of intermediary metabolic products to arginase activation. *The Biochemical Journal*.

[30] Dizikes G. J., Grody W. W., Kern R. M., Cederbaum S. D. (1986). Isolation of human liver arginase cDNA and demonstration of nonhomology between the two human arginase genes. *Biochemical and Biophysical Research Communications*.

[31] Gotoh T., Sonoki T., Nagasaki A., Terada K., Takiguchi M., Mori M. (1996). Molecular cloning of cDNA for nonhepatic mitochondrial arginase (arginase II) and comparison of its induction with nitric oxide synthase in a murine macrophage-like cell line. *FEBS Letters*.

[32] Bagnost T., Ma L., da Silva R. F. (2010). Cardiovascular effects of arginase inhibition in spontaneously hypertensive rats with fully developed hypertension. *Cardiovascular Research*.

[33] Caldwell R. B., Zhang W., Romero M. J., Caldwell R. W. (2010). Vascular dysfunction in retinopathy—an emerging role for arginase. *Brain Research Bulletin*.

[34] Grönros J., Jung C., Lundberg J. O., Cerrato R., Östenson C.-G., Pernow J. (2011). Arginase inhibition restores in vivo coronary microvascular function in type 2 diabetic rats. *American Journal of Physiology—Heart and Circulatory Physiology*.

[35] Grönros J., Kiss A., Palmér M., Jung C., Berkowitz D., Pernow J. (2013). Arginase inhibition improves coronary microvascular function and reduces infarct size following ischaemia-reperfusion in a rat model. *Acta Physiologica*.

[36] Jeyabalan G., Klune J. R., Nakao A. (2008). Arginase blockade protects against hepatic damage in warm ischemia-reperfusion. *Nitric Oxide: Biology and Chemistry*.

[37] Johnson F. K., Johnson R. A., Peyton K. J., Durante W. (2005). Arginase inhibition restores arteriolar endothelial function in Dahl rats with salt-induced hypertension. *The American Journal of Physiology—Regulatory Integrative and Comparative Physiology*.

[38] Ming X. F., Rajapakse A. G., Yepuri G. (2012). Arginase II promotes macrophage inflammatory responses through mitochondrial reactive oxygen species, contributing to insulin resistance and atherogenesis. *Journal of the American Heart Association*.

[39] Morris S. M., Bhamidipati D., Kepka-Lenhart D. (1997). Human type II arginase: sequence analysis and tissue-specific expression. *Gene*.

[40] Ryoo S., Lemmon C. A., Soucy K. G. (2006). Oxidized low-density lipoprotein-dependent endothelial arginase II activation contributes to impaired nitric oxide signaling. *Circulation Research*.

[41] Durante W. (2013). Role of arginase in vessel wall remodeling. *Frontiers in Immunology*.

[42] Jenkinson C. P., Grody W. W., Cederbaum S. D. (1996). Comparative properties of arginases. *Comparative Biochemistry and Physiology B: Biochemistry and Molecular Biology*.

[43] Li H., Meininger C. J., Hawker J. R. (2001). Regulatory role of arginase I and II in nitric oxide, polyamine, and proline syntheses in endothelial cells. *The American Journal of Physiology—Endocrinology and Metabolism*.

[44] Li H., Meininger C. J., Kelly K. A., Hawker J. R., Morris S. M., Wu G. (2002). Activities of arginase I and II are limiting for endothelial cell proliferation. *American Journal of Physiology—Regulatory Integrative and Comparative Physiology*.

[45] Pernow J., Jung C. (2013). Arginase as a potential target in the treatment of cardiovascular disease: reversal of arginine steal?. *Cardiovascular Research*.

[46] Prieto C. P., Krause B. J., Quezada C., San Martin R., Sobrevia L., Casanello P. (2011). Hypoxia-reduced nitric oxide synthase activity is partially explained by higher arginase-2 activity and cellular redistribution in human umbilical vein endothelium. *Placenta*.

[47] Munder M. (2009). Arginase: an emerging key player in the mammalian immune system. *British Journal of Pharmacology*.

[48] Ryoo S., Bhunia A., Chang F., Shoukas A., Berkowitz D. E., Romer L. H. (2011). OxLDL-dependent activation of arginase II is dependent on the LOX-1 receptor and downstream RhoA signaling. *Atherosclerosis*.

[49] Everson W. V., Smart E. J. (2001). Influence of caveolin, cholesterol, and lipoproteins on nitric oxide synthase: implications for vascular disease. *Trends in Cardiovascular Medicine*.

[50] Suschek C. V., Schnorr O., Hemmrich K. (2003). Critical role of L-arginine in endothelial cell survival during oxidative stress. *Circulation*.

[51] Chandra S., Romero M. J., Shatanawi A., Alkilany A. M., Caldwell R. B., Caldwell R. W. (2012). Oxidative species increase arginase activity in endothelial cells through the RhoA/Rho kinase pathway. *The British Journal of Pharmacology*.

[52] Furchgott R. F., Zawadzki J. V. (1980). The obligatory role of endothelial cells in the relaxation of arterial smooth muscle by acetylcholine. *Nature*.

[53] Palmer R. M. J., Ferrige A. G., Moncada S. (1987). Nitric oxide release accounts for the biological activity of endothelium-derived relaxing factor. *Nature*.

[54] Ignarro L. J., Byrns R. E., Buga G. M., Wood K. S. (1987). Endothelium-derived relaxing factor from pulmonary artery and vein possesses pharmacologic and chemical properties identical to those of nitric oxide radical. *Circulation Research*.

[55] Ignarro L. J., Buga G. M., Wood K. S., Byrns R. E., Chaudhuri G. (1987). Endothelium-derived relaxing factor produced and released from artery and vein is nitric oxide. *Proceedings of the National Academy of Sciences of the United States of America*.

[56] Aird W. C. (2007). Phenotypic heterogeneity of the endothelium: I. Structure, function, and mechanisms. *Circulation Research*.

[57] Romero M. J., Platt D. H., Tawfik H. E. (2008). Diabetes-induced coronary vascular dysfunction involves increased arginase activity. *Circulation Research*.

[58] Ludmer P. L., Selwyn A. P., Shook T. L. (1986). Paradoxical vasoconstriction induced by acetylcholine in atherosclerotic coronary arteries. *The New England Journal of Medicine*.

[59] Ross R. (1999). Atherosclerosis—an inflammatory disease. *The New England Journal of Medicine*.

[60] Shimokawa H. (1999). Primary endothelial dysfunction: atherosclerosis. *Journal of Molecular and Cellular Cardiology*.

[61] Lüscher T. F., Tanner F. C., Tschudi M. R., Noll G. (1993). Endothelial dysfunction in coronary artery disease. *Annual Review of Medicine*.

[62] Dhawan V., Handu S. S., Nain C. K., Ganguly N. K. (2005). Chronic L-arginine supplementation improves endothelial cell vasoactive functions in hypercholesterolomic and atherosclerotic monkeys. *Molecular and Cellular Biochemistry*.

[63] Vanhoutte P. M. (2009). How we learned to say no. *Arteriosclerosis, Thrombosis, and Vascular Biology*.

[64] Vanhoutte P. M., Shimokawa H., Tang E. H. C., Feletou M. (2009). Endothelial dysfunction and vascular disease. *Acta Physiologica*.

[65] Zhang C., Hein T. W., Wang W. (2004). Upregulation of vascular arginase in hypertension decreases nitric oxide-mediated dilation of coronary arterioles. *Hypertension*.

[66] El-Bassossy H. M., El-Fawal R., Fahmy A., Watson M. L. (2013). Arginase inhibition alleviates hypertension in the metabolic syndrome. *British Journal of Pharmacology*.

[67] Toque H. A., Nunes K. P., Rojas M. (2013). Arginase 1 mediates increased blood pressure and contributes to vascular endothelial dysfunction in deoxycorticosterone acetate-salt hypertension. *Frontiers in Immunology*.

[68] Hein T. W., Zhang C., Wang W., Chang C.-I., Thengchaisri N., Kuo L. (2003). Ischemia-reperfusion selectively impairs nitric oxide-mediated dilation in coronary arterioles: counteracting role of arginase. *The FASEB Journal*.

[69] Shin W. S., Berkowitz D. E., Ryoo S. W. (2012). Increased arginase II activity contributes to endothelial dysfunction through endothelial nitric oxide synthase uncoupling in aged mice. *Experimental & Molecular Medicine*.

[70] Vaisman B. L., Andrews K. L., Khong S. M. L. (2012). Selective endothelial overexpression of arginase ii induces endothelial dysfunction and hypertension and enhances atherosclerosis in mice. *PLoS ONE*.

[71] Santhanam L., Christianson D. W., Nyhan D., Berkowitz D. E. (2008). Arginase and vascular aging. *Journal of Applied Physiology*.

[72] Madigan M., Zuckerbraun B. (2013). Therapeutic potential of the nitrite-generated NO pathway in vascular dysfunction. *Frontiers in Immunology*.

[73] Galle J., Bengen J., Schollmeyer P., Wanner C. (1995). Impairment of endothelium-dependent dilation in rabbit renal arteries by oxidized lipoprotein(a): role of oxygen-derived radicals. *Circulation*.

[74] Böger R. H., Sydow K., Borlak J. (2000). LDL cholesterol upregulates synthesis of asymmetrical dimethylarginine in human endothelial cells: involvement of S-adenosylmethionine-dependent methyltransferases. *Circulation Research*.

[75] Ryoo S., Berkowitz D. E., Lim H. K. (2011). Endothelial arginase II and atherosclerosis. *Korean Journal of Anesthesiology*.

[76] Brunner H., Cockcroft J. R., Deanfield J. (2005). Endothelial function and dysfunctio, part II: association with cardiovascular risk factors and diseases: a statement by the Working Group on Endothelins and Endothelial Factors of the European Society of Hypertension. *Journal of Hypertension*.

[77] Witztum J. L., Steinberg D. (2001). The oxidative modification hypothesis of atherosclerosis: does it hold for humans?. *Trends in Cardiovascular Medicine*.

[78] Steinberg D., Witztum J. L. (2002). Is the oxidative modification hypothesis relevant to human atherosclerosis? Do the antioxidant trials conducted to date refute the hypothesis?. *Circulation*.

[79] Yan G., You B., Chen S.-P., Liao J. K., Sun J. (2008). Tumor necrosis factor-*α* downregulates endothelial nitric oxide synthase mRNA stability via translation elongation factor 1-*α* 1. *Circulation Research*.

[80] Spillmann F., van Linthout S., Miteva K. (2014). LXR agonism improves TNF-*α*-induced endothelial dysfunction in the absence of its cholesterol-modulating effects. *Atherosclerosis*.

[81] Witting P. K., Song C., Hsu K. (2011). The acute-phase protein serum amyloid A induces endothelial dysfunction that is inhibited by high-density lipoprotein. *Free Radical Biology & Medicine*.

[82] Thacher T. N., Gambillara V., Riche F., Silacci P., Stergiopulos N., da Silva R. F. (2010). Regulation of arginase pathway in response to wall shear stress. *Atherosclerosis*.

[83] Xiong Y., Yu Y., Montani J.-P., Yang Z., Ming X.-F. (2013). Arginase-II induces vascular smooth muscle cell senescence and apoptosis through p66Shc and p53 independently of its l-arginine ureahydrolase activity: implications for atherosclerotic plaque vulnerability. *Journal of the American Heart Association*.

[84] Ming X.-F., Barandier C., Viswambharan H. (2004). Thrombin stimulates human endothelial arginase enzymatic activity via RhoA/ROCK pathway: implications for atherosclerotic endothelial dysfunction. *Circulation*.

[85] Teupser D., Burkhardt R., Wilfert W., Haffner I., Nebendahl K., Thiery J. (2006). Identification of macrophage arginase I as a new candidate gene of atherosclerosis resistance. *Arteriosclerosis, Thrombosis, and Vascular Biology*.

[86] Steppan J., Nyhan D., Berkowitz D. E. (2013). Development of novel arginase inhibitors for therapy of endothelial dysfunction. *Frontiers in Immunology*.

[87] Fraga-Silva R. A., Costa-Fraga F. P., Faye Y. (2014). An increased arginase activity is associated with corpus cavernosum impairment induced by hypercholesterolemia. *Journal of Sexual Medicine*.

[88] El-Bassossy H. M., El-Fawal R., Fahmy A. (2012). Arginase inhibition alleviates hypertension associated with diabetes: effect on endothelial dependent relaxation and NO production. *Vascular Pharmacology*.

[89] Gonon A. T., Jung C., Katz A. (2012). Local arginase inhibition during early reperfusion mediates cardioprotection via increased nitric oxide production. *PLoS ONE*.

[90] White A. R., Ryoo S., Li D. (2006). Knockdown of arginase I restores NO signaling in the vasculature of old rats. *Hypertension*.

[91] Kim J. H., Bugaj L. J., Oh Y. J. (2009). Arginase inhibition restores NOS coupling and reverses endothelial dysfunction and vascular stiffness in old rats. *Journal of Applied Physiology*.

[92] Jung C., Figulla H. R., Lichtenauer M., Franz M., Pernow J. (2014). Increased levels of circulating arginase I in overweight compared to normal weight adolescents. *Pediatric Diabetes*.

[93] Holowatz L. A., Kenney W. L. (2007). Up-regulation of arginase activity contributes to attenuated reflex cutaneous vasodilatation in hypertensive humans. *The Journal of Physiology*.

[94] Holowatz L. A., Santhanam L., Webb A., Berkowitz D. E., Kenney W. L. (2011). Oral atorvastatin therapy restores cutaneous microvascular function by decreasing arginase activity in hypercholesterolaemic humans. *Journal of Physiology*.

[95] Woo A., Min B., Ryoo S. (2010). Piceatannol-3′-O-*β*-D-glucopyranoside as an active component of rhubarb activates endothelial nitric oxide synthase through inhibition of arginase activity. *Experimental & Molecular Medicine*.

[96] Ramírez-Zamora S., Méndez-Rodríguez M. L., Olguín-Martínez M. (2013). Increased erythrocytes by-products of arginine catabolism are associated with hyperglycemia and could be involved in the pathogenesis of type 2 diabetes mellitus. *PLoS ONE*.

[97] Shin W., Cuong T. D., Lee J. H. (2011). Arginase inhibition by ethylacetate extract of Caesalpinia sappan lignum contributes to activation of endothelial nitric oxide synthase. *The Korean Journal of Physiology and Pharmacology*.

[98] Joe Y., Zheng M., Kim H. J. (2012). Salvianolic acid B exerts vasoprotective effects through the modulation of heme oxygenase-1 and arginase activities. *Journal of Pharmacology and Experimental Therapeutics*.

